# Supplementary material for: Effectiveness and experiences with differentiated service delivery of HIV care in Kisumu County, Kenya: A mixed methods study, 2014–2021
Source: PLOS Glob Public Health. 2025 Aug 1;5(8):e0004481. doi: 10.1371/journal.pgph.0004481 (PMC12316313; doi:10.1371/journal.pgph.0004481)
Supplement: S1 Table — (DOCX) [file pgph.0004481.s001.docx]

**S1 Table: Relative Risk analysis of retention, overall and stratified by DSD track, gender, and age**

| **Retention** | **Baseline*** | **Midline** | **R. R** | **Endline** | R. R |
| --- | --- | --- | --- | --- | --- |
|  | **N (%)** | **N (%)** | **95% C. I** | **N (%)** | **95% C. I** |
|  |  |  |  |  |  |
| **Sex** |  |  |  |  |  |
| **Male** | 7,513 (85.6) | 5,461 (99.1) | 1.015 (1.005-1.026) | 2336 (98.6) | 1.152(1.141-1.163) |
| **Female** | 13,454 (86.9) | 10125 (98.9) | 1.138 (1.131-1.145) | 4296 (98.9) | 1.138(1.130-1.146) |
|  |  |  |  |  |  |
| **Age** |  |  |  |  |  |
| **20-34 years** | 9,920 (81.4) | 5275 (98.6) | 1.211 (1.202-1.221) | 2381 (98.7) | 1.213 (1.201-1224) |
| **35-49 years** | 8,150 (91.2) | 7227 (99.1) | 1.087 (1.079-1.094) | 3014 (99.1) | 1.087 (1.079-1.095) |
| **50+ years** | 2,897 (92.5) | 3084 (99.3) | 1.073 (1.062-1.085) | 1237 (98.6) | 1.066 (1.053-1.078) |
|  |  |  |  |  |  |
| **Baseline WHO Staging** |  |  |  |  |  |
| WHO Stage 1 | 8,797 (84.8) | 4,443 (99.5) | 1.173 (1.164-1.183) | 1,792 (98.8) | 1.007 (1.002-1.013) |
| WHO Stage 2 | 6,663 (90.6) | 3,736 (99.7) | 1.100 (1.092-1.109) | 1,429 (99.0) | 1.093 (1.083-1.103) |
| WHO Stage 3 | 4,329 (91.9) | 2,577 (99.7) | 1.085 (1.075-1.094) | 1189 (99.3) | 1.081 (1.071-1.091) |
| WHO Stage 4 | 864 (92.8) | 557 (99.1) | 1.068 (1.047-1.089) | 171 (98.8) | 1.065 (1.04-1.09) |
|  |  |  |  |  |  |
| **Components of the package of ART distribution options** | | | | |  |
| **Community ART Groups [CAGs]** | n/a | 228 (100) |  | 27 (100) | - |
| **FastTrack ART/Express** | n/a | 11,026 (99.6) |  | 4,532 (99.0) | 0.994 (0.991-0.997) |
| **Facility-based ART Groups [FB-AG]** | n/a | 59 (95.2) |  | 22 (95.7) | 1.005 (0.9906-1.115) |
| **Standard of Care [SOC]** | 20,967 (86.4) | 4,273 (98.3) | 1.138 (1.130-1.145) | 2,051(98.5) | 1.140 (1.32-1.148) |
| **All the relative risk analysis are done using the baseline as the reference.* | | |  |  |  |
