# Supplementary material for: Effectiveness and experiences with differentiated service delivery of HIV care in Kisumu County, Kenya: A mixed methods study, 2014–2021
Source: PLOS Glob Public Health. 2025 Aug 1;5(8):e0004481. doi: 10.1371/journal.pgph.0004481 (PMC12316313; doi:10.1371/journal.pgph.0004481)
Supplement: S1 File — (DOCX) [file pgph.0004481.s004.docx]

S1 File: Differentiated Care Focus Group Discussion Guide

Patient Guide

Purpose: To understand patient experiences with HIV differentiated care, the challenges, benefits, satisfaction, and recommendations for improvement.

INTRODUCTION

My name is ___________________. I am working with Family AIDS Care and Education Services

(FACES), a project aiming to improve the lives of people living with HIV. Thank you for taking the time

to talk today. I would like to talk to you about differentiated care services. I would like

to hear about your experiences with differentiated care and how it has or has not impacted your care and wellbeing.

Please keep the information discussed in the focus group discussion (FGD) is confidential. No identifying information will be shared with health care providers, your privacy is important to us. No

one’s care will be affected by information shared in this discussion. The information that you provide

will be used to inform efforts to strengthen and improve health services in Kisumu

County. This FGD will take around one hour and half hours. If you have questions you want to ask

about on other topics, I can assist you to find answers after the focus group discussion is over. In order

not to lose any of the valuable information you tell me, I will record our conversation. After a

transcript of the audio recording is typed up, the recording will be destroyed. Your name will not

be attached to the recording or the transcript, and only members of the study team will be

permitted to listen to what you say. Before deciding let’s go through the consent form (proceed

through informed consent).

(If there is agreement for tape recording and consent is obtained, you may start recording)

1. Tell me about your understanding of HIV differentiated services?

*(Provide differentiated care overview after asking the first question)*

| HIV differentiated services cater to stable patients on ART. The service includes less frequent clinic appointments for clinical review, with ART and other chronic medicines refilled through a distribution system in between clinical review visits. This is to reduce the burden on patients and the health facility. There are a few different models of differentiated care.  **Facility-based Fast Track (FAST TRACK)** – patients come to clinic every six months for a clinical review appointment, with ART refill visits in-between, every three months.  **Community ART Groups (CAG)** – patients come to the clinic every six months for a clinical review appointment, with ART refills are distributed through a support-group structure through a community ART group in between clinic visits, every three months.  **Peer-led Community ART Groups (Peer-led CAG)** – A peer-led community ART group meets monthly in the community. Each peer community ART group member comes to the clinic every six months for a clinical review appointment. During that visit, the member picks up a one-month supply of pre-packed and labeled ART for themselves and the other group members. During the monthly meeting, attendance and health are assessed and documented to check on stable eligibility and to facilitate referral to the facility if needed. ART is then distributed by the member who was assigned to pick up the groups’ ART at their last clinic visit.  **Healthcare Worker-led Community ART Groups (HCW CAG)** This model is similar to the peer-led model. However, the health care worker attends every community meeting and facilitates the ART distribution and documentation. |
| --- |

- 1. Are any of these approaches new information to you?
  2. Where have you learned about the differentiated care options (probe: from where and whom within the clinic [eg. health talk, clinician, poster)
  3. Tell me about the differentiated care model you are currently in?

1. Thinking back to when you first started in differentiated care..
   1. How did the clinic explain it to you?
   2. Was it offered as a choice?
2. A new or different way of receiving services may impact how you access or receive care, it may bring advantages or personal challenges and may affect you in other ways.
   1. How has differentiated care impacted your HIV care in positive or beneficial ways?
   2. Please tell me about any challenges or barriers to HIV care you may have experienced with differentiated care?
   3. How has it impacted your health, for instance your ability to adhere to treatment and your viral load?
   4. How has it impacted your well-being?
3. Please tell me more about your experiences with differentiated care?
   1. How well does the flow or process work during refill pick up or distribution?
   2. What are your interactions with pharmacy or community group leaders like during drug pick up or distribution?
   3. What are your interactions with providers like during clinical visits?
   4. How has the clinic changed since differentiated care services started, what have you noticed?
   5. How have differentiated care services changed during COVID-19?
   6. How might you improve HIV differentiated care services?
   7. Overall, how satisfied would you say you are with differentiated care services that you are currently in?
4. Have there been opportunities to shift to other models of differentiated care?
   1. If you have shifted between differentiated models, what motivated you to change?
   2. What was the transition like, any challenges or concerns?
5. From the DSD models that we have described, is there a preferred model? Please tell me why?
6. From the described DSD models, please tell me about those that may be available, but not accessible to you and why?
7. Are there differentiated service models that are needed and are unavailable? Why are they difficult to access?
8. Is there anything else you would like to add before we close?

Thank you for your time. Your responses will be very helpful for improving service delivery.
