# Supplementary material for: Effectiveness and experiences with differentiated service delivery of HIV care in Kisumu County, Kenya: A mixed methods study, 2014–2021
Source: PLOS Glob Public Health. 2025 Aug 1;5(8):e0004481. doi: 10.1371/journal.pgph.0004481 (PMC12316313; doi:10.1371/journal.pgph.0004481)
Supplement: S2 File — (DOCX) [file pgph.0004481.s005.docx]

S2 File: Differentiated Care Focus Group Discussion Guide

Health Care Worker Guide

Purpose: To understand health care worker perspectives and experiences with HIV differentiated care, the challenges, benefits, impact, and recommendations for improvement.

INTRODUCTION

My name is ___________________. I am working with FACES to learn more about your perspectives and experiences with differentiated care services. I would like to hear about your experiences with differentiated care, how it is going, how you feel it has impacted your work, patient care, and the health facility in general. Thank you for taking the time to talk today.

Please keep the information discussed in the focus group discussion (FGD) is confidential. No identifying information will be shared outside of this group, your privacy is important to us. Your work will be not be affected by information shared in this discussion. The information that you provide

will be used to inform efforts to strengthen and improve health services in Kisumu

County. This FGD will take around one hour and half hours. If you have questions you want to ask

about on other topics, I can assist you to find answers after the focus group discussion is over. In order

not to lose any of the valuable information you tell me, I will record our conversation. After a

transcript of the audio recording is typed up, the recording will be destroyed. Your name will not

be attached to the recording or the transcript, and only members of the study team will be

permitted to listen to what you say. Before deciding let’s go through the consent form (proceed

through informed consent).

(If there is agreement for tape recording and consent is obtained, you may start recording)

1. Role in Differentiated Service Delivery (DSD)
   1. How would you describe your comfort level supporting clients on HIV Differentiated Service Delivery?
   2. Which Differentiated Service Delivery model do your clients utilize the most? and why?
   3. Which Differentiated Service Delivery models are critical in addressing your clients` needs and why?
   4. Which Differentiated Service Delivery models do your clients need and are unable to access?
      1. What are the reasons they cannot access them?
   5. What are the Differentiated Service Delivery needs that are specific to a certain population such as?
      1. Pregnant women
      2. Adolescents
      3. Children
      4. Youth 20 to 24-year-old
      5. Clients in formal employment
   6. How has differentiated care impacted your daily work?
   7. How has differentiated care services impacted the health facility (probe: CCC/PSC)?
   8. How have differentiated care services for patients been impacted by COVID-19?
2. Benefits of Differentiated Service Delivery (DSD)
   1. What do clients like about Differentiated Service Delivery , what benefits do they mention about facility-based and community-based Differentiated Service Delivery?
      1. Pregnant women
      2. Children
      3. Adolescents
      4. Youth
      5. Clients In formal employment
3. Challenges and barriers to Differentiated Service Delivery (DSD).
   1. What are the common concerns your clients have about Differentiated Service Delivery ?
   2. Thinking about the clinical review, are there specific concerns or challenges clients experience related to clinic review appointments? (probe: appointment times, getting to the clinic, waiting times, clinic operating hours, provider interaction, and clinic flow)?
   3. Thinking about ART refill visits at the facility, are there specific concerns or challenges clients experience related to ART refill visits? (probe: scheduling, wait times, clinic flow, staff interaction)
   4. Thinking about distribution points in the community, are there specific concerns or challenges clients experience related to ART distribution? (probe: structure, process, reliability, confidentiality)
   5. What unique challenges have you noticed with specific populations such as?
      1. Pregnant women
      2. Adolescents
      3. Children
      4. Youth
      5. Clients in formal employment
4. From your experience, how does Differentiated Service Delivery impact patient health behaviors and outcomes (probe: access to care, adherence/retention, viral load)?
5. How might we improve Differentiated Service Delivery, what should be prioritized for improvement?
6. Any additional thoughts, suggestions, or ideas you would like to share with us at this time?

Thank you for your time. Your responses will be very helpful for improving service delivery.
