# Supplementary material for: Effectiveness and experiences with differentiated service delivery of HIV care in Kisumu County, Kenya: A mixed methods study, 2014–2021
Source: PLOS Glob Public Health. 2025 Aug 1;5(8):e0004481. doi: 10.1371/journal.pgph.0004481 (PMC12316313; doi:10.1371/journal.pgph.0004481)
Supplement: Attachment — Submitted filename: Response to Reviewers_16May2025.docx [file pgph.0004481.s006.docx]

Raphael Onyango Mando KEMRI-RCTP

P. O. B 614 -40100 KISUMU

Email: raphaelmando@icloud.com

14th February 2025

The Editor, PLOS

1875 Mission Street

Suite 103 #188 San Francisco, CA 94103

United States

Dear Editor,

On behalf of the co-authors, I am pleased to submit the revised manuscript addressing the review comments of Reviewer 1 and reviewer 2 on the manuscript entitled “Eﬀectiveness and Experiences with Diﬀerentiated Service Delivery of HIV Care in Kisumu County, Kenya: A Mixed Methods Study, 2014-2021.” This manuscript delves into the concept of diﬀerentiated service delivery and its implications within the context of HIV care.

Please find our responses below.

### **Effectiveness and experiences with differentiated service delivery of HIV care in Kisumu County, Kenya: A mixed methods study, 2014-2021**

Response to Reviewers

Reviewer # 1

Introduction

**1.** The literature to back up the study rationale is lacking. There are many studies that describe clinical outcomes of DSD in adults living with HIV as well as client and HCW experiences. It would be useful to the reader to add some literature on clinical outcomes and client/ health care worker experiences from Sub-Saharan Africa and Kenya (if there are any) and then articulate the research gap more clearly.

**Response**: Thank you for pointing this out. We have added more content and literature to the background, lines 67-74 &77-80.

Methods

**2**. Outcomes- why was a viral load cut off of <1000 copies/ml selected as the viral suppression outcome? Did the authors consider using an additional more stringent cut off such as <50 copies/ml in their analysis? Can the authors also elaborate more on the monitoring criteria used for viral suppression from the local Kenyan DSD guideline, and how this influenced the viral load cut off used in the study.

**Response**: At the time of the analysis the national guidelines recommended that the cut off for viral suppression as <1000 copies/ml. Currently the cut off is <200 copies/ml. The National HIV Care and Treatment Guidelines recommend that each individual adult have at least one viral load test as part of the routine care package at least every 12 months.

**3.** The authors need to clarify the time point that was used to define a 12-month viral load and a 24-month viral load. In clinical practice, viral loads are generally not taken at the specified time point. Can the authors clarify what the 12-month window and 24-month window was used for the viral suppression outcome.

**Response:** We extracted viral load data for the baseline period October 2015-September 2016 and for the midline period between October 2017 - September 2018 and for the endline period between October 2020 - September 2021. In case an individual had two viral data points we kept the last valid viral data point. We have added the text in line 147-150 of the manuscript.

Ethics

**4**. The authors state that a waiver was given not to obtain informed consent. Was this for the electronic data only? Why was no written informed consent obtained for the focus group discussions? Focus group discussions cannot be considered to be part of routine care.

**Response**: Yes, the waiver of consent was strictly for the electronic data. We obtained informed consent for the focused group discussions and have edited the ethics statement accordingly. We also moved the ethics statement to the end of the Methods section (see lines 253-258).

Data collection

**5**. The description of the study methodology for the qualitative component is missing some important components. It is not clear what methodology was employed- thematic analysis, framework analysis, grounded theory. It is also not clear what approach was taken in analyzing data, an inductive or deductive approach? How were coding discrepancies resolved? How was the accuracy of translation achieved for the non-English transcripts?

To enhance transparency and rigor of the manuscript, can the authors please provide a copy of the focus group interview guide used for this study as a supplementary file?

**Response:** We have strengthened the qualitative data collection and data analysis sections to provide more details on codebook development process, how data was verified, and the analytical approach carried out. We have included the guides as supplemental files as suggested. See lines 202-203 and lines 227-239.

Data analysis

**6**. It is unclear how the exposure variables were selected. Can the authors elaborate more on how the exposure variables for the logistic regression model were selected?

**Response:** This has currently changed because we finally used relative risk for the groups to report this outcome.

**7.** The authors need to make it more clear how they calculated the retention post DSD enrolment, more specifically what exclusions were applied. From figure 2, it looks like those who did not make it to the 12 and 24-month follow-up were excluded from the analysis, but this is not articulated in the methods section.

**Response:** Individuals who had not matured in care were excluded in the final analysis at month 12 and 24 in care. Maturity in care essentially means that for an individual to be eligible for evaluation whether to be retained or not at month 12 and 12 respectively. They ought to have been in DSD for at least 12 months at the time of evaluation based on the time when they were enrolled into any DSD model. This is clarified in the paper, see lines 116.

Results

**8.** Line 203- Please add the percent symbol after 86.4.

**Response:** We have added the percent, it now reads 86.4%, see line 263.

**9.** Table 1, 2 and 3 are missing from the manuscript file. The results are referenced in the text, but the tables are missing.

**Response**: These were initially uploaded separately as independent file. They have now been added to the main manuscript.

**10.** Line 226. The authors have given the viral suppression proportions at midline and endline. Did all clients have a viral load done? It seems unlikely that 100% of clients would have viral loads done at 12 and 24-month mark. What were the proportions for viral load completion at baseline, midline and endpoint? The denominator for viral load suppression would need to exclude those who did not have a viral load done.

**Response**: We analyzed viral load suppression data only for clients that had viral load done. The data for the viral load data analysis used here was sourced separately form the national viral load website (https://nascop.org) and so vl data includes only those with some viral load outcome. Please view our table on viral load analysis. See line 150-153.

**11.** The authors have conducted a multivariable logistic regression analysis for retention at baseline, 12 months post DSD enrollment, and 24 months post DSD enrolment. The data on factors associated with viral suppression have not been presented. Can the authors consider adding this as part of their analysis as a supplementary table?

**Response:** We appreciate this suggestion, however regressing the viral suppression numbers against sociodemographic may not add new insights to information already out there and the fact that the second reviewer is proposing that we further segregate the suppression at <1,000 copies/mL further into smaller subcategories. Moreover, we no longer have the funding to carry out any additional analysis for this manuscript*.*

Discussion

**12**. Are there any DSD studies that have been previously published from the Kenyan setting for comparison purposes to the current study?

**Response**: We have added DSD studies from the local context, which are now cited in the background.

**13**. The study produced some interesting insights on recommendations for improvement of DSD. However, it would be useful if the authors could suggest some practical strategies to mitigate challenges raised such as privacy and preference for longer ART refills/less clinic visits.

**Response:** This has been addressed (community clubs with locations selected based on privacy show promise), with literature added, see line 569-570.

**14.** One major limitation that the authors need to consider is the lack of a valid control group due to the before/after study design used. Therefore, there are some confounders that could not be adjusted for in the regression analysis such as ART policy changes (such as introduction of dolutegravir) and COVID-19 that would have had an impact on viral suppression and retention.

**Response:** The uptake of DTG at the end of the evaluation period was 72% (June 2021) while at the start of the evaluation period (Baseline) the uptake of DTG was quite low at 2.1%. The good retention observed here at least in the baseline cannot be wholly attributed to ART policy changes (such as introduction of dolutegravir). In fact, differentiated service delivery was a better way of dealing with the challenges of COVID-19 due to longer spacing between visits and other elements that it came with. We have indicated that DTG may have played a role, see lines 486-490 and in the limitations we have noted the lack of a control group and discussed other factors that may have been confounders, see lines 578-594.

**15.** What are the strengths of this study?

**Response:** We have added this to the manuscript, prior to the limitations. “This study’s strengths include utilizing a large sample size from a routine setting, patient level data and a long duration of follow-up to examine clinical outcomes in the DSD context. This study provides insights on the effectiveness of DSD models on sustained retention on ART and viral suppression.” See lines 578-581.

**Reviewer #2:**

Thank you for the opportunity to review the manuscript entitled “Effectiveness and experiences with differentiated service delivery of HIV care in Kisumu County, Kenya: A mixed methods study, 2014-2021”. Key strengths include the large sample size and duration of study observation. I also appreciate the mixed method approach and the results from the qualitative findings were insightful.

**16**. My main concerns are methodologic; while DSD outcomes are notoriously difficult to estimate due to difficulty establishing a true counterfactual that can stand in as a comparison group for those enrolled in DSD models, the authors have not appropriately addressed this analytic challenge here. Much of the approach to definitions and data analysis is unclear from the manuscript text. In addition, the interpretation of the study findings which directly attribute the unusually high retention outcomes directly to the implementation of DSD needs addressing, especially given the noted potential for bias. I have several comments for the authors detailed below which should be address before publication could be considered:

**Response:** In our study design we have a period before the roll out of DSD, pre-DSD (before implementation of DSD) where we used the existing data to assess if the clients mimicked DSD even though DSD was not available during that time. This included a six steps procedure where individuals must have (i). been in care for the past 12 months, (ii). Have had IPT for 6 Months, (iii). To having tuberculosis (iv). virally suppressed, (v). not having any other opportunistic infections and (vi). not having any adherence issues with the facility based on the clinician’s assessment. We then assessed their retention status. And then compared that to the 12-month retention of those who were explicitly on DSD. Nevertheless, we do agree that the lack of a comparison group is a limitation and we have added this to the limitations, along with the role of possible confounders.

*Major comments:*

17. Introduction – the expanse of existing DSD literature is not well covered. The statement “there is limited data on the effectiveness of DSD models as well as patient and provider perceptions of these approaches” seems unfounded – in fact, there are extensive publications around effectiveness and acceptability of DSD approaches in sub-saharan Africa (see authors Bolton-Moore, Bwire, Mukumbang, Mokhele, Huber, Pascoe, Long etc.) These should be explored and a more balanced perspective brought to the background.

**Response:** Thank you for pointing this out. We have omitted that sentence and added more content and literature to the Introduction.

18. The primary retention outcome seems problematic – currently it is defined as attendance at one visit in a 2-month window over the course of 12 or 24 months. This hardly seems like an accurate picture of retention in care. A client could simply have re-engaged in care and attended a visit in that period but missed several medication pickups during the course of the year. Given the data source available to the authors, alternate retention outcomes such as disengagement from care or treatment interruptions (missing at least one clinic visit by >28 days for example as per PEPFER definitions) should be considered.

**Response:** Our definition of retention is being in care for 12 months -1/+ more after being enrolled into DSD which is based on the last date of visit at the facility and not, “one visit in a 2-month window over the course of 12 or 24 months” as suggested. See details in line 139-140 in the measurements section of the manuscript.

19. The methods text describing person time in the analysis isn’t clear – what is meant by “people could enter the cohort at any time”?

**Response**: What we mean is that individuals might have been transferred into these facilities or enrolled at some time during the process of evaluation and got eligible during the process.

20. How were outcomes ascertained if a person hadn’t been on a DSD model for 12 months?

**Response:** Individuals who had not been on the DSD model were considered not retained.

21. Was transfer exclusively to another facility?

**Response:** Transfer outs were removed from the denominator outrightly.

22. How did decanting back to non-DSD model affect analysis? Were those included in outcome “retained”?

**Response:** Since their data was part of the larger initial dataset, we were able to tell their outcome based on their last visit at the facility.

23. For the pre-baseline group, their person-time for the primary outcome needs to be better clarified. We assume they entered the cohort when they met eligibility criteria for DSD enrolment but when was their outcome defined? Was it 12 months after they met eligibility criteria?

**Response:** Yes, their outcome was defined 12 months post eligibility.

24. The statistical methods are not wholly appropriate here. My main concern is with the use of logistic regression and odds ratios to present the key study findings – differences in risk between the three exposure groups. **As the authors have the population denominator in these data, risk differences and relative risks with 95% CI can be calculated.** The relative risk is what you are actually after in this scenario (probability of the event occurring in all exposed individuals versus the event occurring in all non-exposed individuals). Odds ratios tend to overestimate true relative risk with common outcomes; the overestimation becoming worse the more common the outcome (as in this case where the chosen outcome is >80% across all groups). This may be contributing to the very over-inflated OR estimates presented in the results and should be avoided in this case. I would suggest to rather estimate and present risk differences and relative risks (risk ratios) with 95% CI. You can then also use your confidence intervals to determine meaningful differences between groups rather than p values which should be reserved for hypothesis testing (also not what the authors are trying to achieve here).

**Response:** The table has been revised to reflect the relative risk and the results table updated effectively.

25. Who is the group “imputed” to be on DSD? Is this the baseline group? If someone met the criteria for eligibility for DSD but wasn’t observed in the data to be in DSD care, that doesn’t mean they were. They could have simply refused to be in a DSD model and would be very different from those who opted to enroll?

**Response:** These are individuals whose DSD status were not marked in the dataset yet based on the available indicators we were able to tell whether they were on a differentiated care model. For instance, we could tell based on the period between two visit dates was long enough to warrant them being on some DSD model.

26. The presentation of the outcomes seems incomplete. I have concerns with excluding those who elected not to be enrolled in DSD from retention calculations. The comparison group for the “baseline” include all who were eligible for DSD whether they would have enrolled or not. The same population should be included or at least presented for all groups – currently the outcomes for those who elected not to be in DSD are not shown.

**Response:** The baseline table looks so because we used the available six steps for evaluation for eligibility for enrollment into DSD to pick only those whose characteristics emulated DSD and evaluated their retention numbers. Those who elected not to be on any DSD remained in Standard of Care and are part of the analysis as shown in the tables.

27. Moreover, outcomes of those not retained should be clarified – numbers transferred, deceased, dropped out of care, or became unstable. I think it’s in Figure 2 but its so illegible, it could be helpful in the text.

**Response:** We have made the text legible in the table. Please review to confirm**.**

28. The sensitivity analysis results need further explanation – in the methods they are indicated that these analyses would be to justify the inclusion of the ‘imputed” DSD group into the noted DSD group, yet no outcomes are presented for this group and the concept of a gap between clinical encounters of more than 6 months is described in the results (has never been reported or described prior to this point in the manuscript). This should be explained and justified in the methods.

**Response:** We have reviewed this section of the manuscript and confirmed that the process conducted was an imputation and not a sensitivity analysis. The analysis presented was used to assess if the patients whose periods between visits dates mimics those of differentiated service delivery model which in this case was about 180 days hence, they were moved to the rightful category. The purpose of this was to support the decision to collapse that group with those noted explicitly as being in DSD. We have added the justification description in the Methods to cover this analysis in the methods*.* See line 187 to 189.

29. The viral load suppression results presented were very limited. There is no text describing which VL tests were included and over which time periods in the methods nor any information on how missing VL test results were handled. Current interest in low-grade viremia offers an opportunity for some interesting analyses if the definition of suppressed VL (currently defined at <1000 copies/mL) were further stratified into 1) VL suppressed (<50 copies/mL), 2) low-grade viremia (50-1000 copies/mL) and 3) high grade viremia (>1000 copies*/mL).*

**Response:** In the Methods, we have added content on the viral load tests, time periods and how the missingness was handled. See line 147 to153.

We appreciate the suggestion to explore low grade viremia and stratify into suppressed, low grade, and high grade viremia, however we no longer have the funding to carry out any additional analyses.

30. The text describing the multivariable analysis could use some revision for clarity; particularly with respect to which group and time is being used as the reference group.

**Response:** We have changed the analysis to relative risk. Therefore, this comment no longer applies.

31. The focus group discussion results offer helpful insights and are a valuable contribution to the manuscript. They are however, somewhat one-sided in focusing on the benefits of the DSD models among those using them. The insights among those who were eligible but elected not to enroll in DSD models should also be presented; these insights are key to understanding challenges with uptake or implementation of DSD.

**Response:** Thank you for pointing this out. We did not conduct focus groups among those who did not choose to participate in DSD. We have added this to the limitations, see line 597. We also worked further on interpretation of the qualitative findings in the Discussion with substantiated literature.

32. One of my key concerns is that the interpretation of the effect of DSD models on retention outcomes needs to be tempered. Retention rates are unusually high and direct comparisons with other published estimates from the region are missing from this manuscript. Currently, the text attributes the improvement in retention outcomes directly to DSD implementation. However, the authors note an important source of bias in this regard in the discussion – the program-wide intensified retention strategies carried out during this evaluation period certainly would have impacted on estimates of retention and create further problems with finding a true comparison group for the DSD enrolled group. This is not addressed in the limitations section at all. Sensitivity analyses could explore the impact this may be having but the sensitivity analyses presented are limited and confusing as to what they are trying to achieve.

**Response:** We appreciate this comment. We have tempered the interpretation of the results and conclusion, discussed other possible explanations, added more literature and greatly improved the limitations. See lines 461-492, 583-594, and 601-602.

33. The VL suppression outcomes have a similar problem in terms of attributing the improvements to DSD while other factors (DTG rollout, all the retention interventions introduced etc) would have made an important impact on numbers suppressed.

**Response (same as response to #14)**: The uptake of DTG at the end of the evaluation period was 72% (June 2021) while at the start of the evaluation period (Baseline) the uptake of DTG was quite low at 2.1%. The good retention observed here at least in the baseline cannot be wholly attributed to ART policy changes (such as introduction of dolutegravir). In fact, differentiated service delivery was a better way of dealing with the challenges of COVID-19 due to longer spacing between visits and other elements that it came with. We have indicated that DTG may have played a role, see lines 486-490 and in the limitations, we have noted the lack of a control group and discussed other factors that may have been confounders, see lines 578-594.

Minor comments:

34. Page 3 line 62: Sentence does not make sense – “Poor retention in HIV care and treatment programs has been identified as the most common reason for treatment failure among PLHIV on ART (14,15), with losses to follow up being a key driver of poor retention.” Loss to follow up is the same as poor retention? And not being on treatment could only be considered treatment failure surely?

**Response**: We have edited this sentence for clarity. See line 63.

35. On the health care worker side, more time focused on managing complex cases and bolstering quality of care likely not only benefited clients enrolled in DSD models but also outcomes clinic wide” – could you expand on what the clinic-wide outcomes would be that would improve?

**Response**: This has been revised for clarity. “On the health care worker side, more time focused on managing complex cases and bolstering quality of care was likely not only a benefit to clients enrolled in DSD models but also benefited health care workers who felt less rushed and fatigued, had better attitudes, and more time to manage clients.”, see lines 503-505.

36. The behavioral theories noted in the discussion are great – these should be cited though and examples cited of this in action in other studies examining health outcomes to illustrate how this applies to DSD implementation.

**Response:** This is noted, content and literature has been added. See lines 538-558

37. The figures (especially figure 2) are almost completely illegible and make understanding results very difficult.

**Response:** These have been converted using the journal recommended Preflight Analysis and Conversion Engine (PACE) digital diagnostic tool, <https://pacev2.apexcovantage.com/> to more legible formats. You may also need to zoom in a little bit.

38. Suppl table multivariate outcomes need to report N achieving each outcome by each stratification level.

**Response:** The table has been revised to relative risk and reflects the absolute numbers reported in each category. Check question 5 on the major comments because this question may walk back on the major comments.

Sincerely,

Raphael.

//
